# Quantum Image Encryption Scheme Using Arnold Transform and S-box Scrambling

**DOI:** 10.3390/e21040343

**Published:** 2019-03-28

**Authors:** Hui Liu, Bo Zhao, Linquan Huang

**Affiliations:** 1 Key Laboratory Aerospace Information Security and Trusted Computing, Ministry of Education, School of Cyber Science and Engineering, Wuhan University, Wuhan 430072, China; 2 School of Computer Science and Technology, Hankou University, Wuhan 430212, China

**Keywords:** S-box scrambling, mutation operation, Arnold transform, SHA-256 hash, quantum chaotic map

## Abstract

The paper proposes a lossless quantum image encryption scheme based on substitution tables (S-box) scrambling, mutation operation and general Arnold transform with keys. First, the key generator builds upon the foundation of SHA-256 hash with plain-image and a random sequence. Its output value is used to yield initial conditions and parameters of the proposed image encryption scheme. Second, the permutation and gray-level encryption architecture is built by discrete Arnold map and quantum chaotic map. Before the permutation of Arnold transform, the pixel value is modified by quantum chaos sequence. In order to get high scrambling and randomness, S-box and mutation operation are exploited in gray-level encryption stage. The combination of linear transformation and nonlinear transformation ensures the complexity of the proposed scheme and avoids harmful periodicity. The simulation shows the cipher-image has a fairly uniform histogram, low correlation coefficients closed to 0, high information entropy closed to 8. The proposed cryptosystem provides 2^256^ key space and performs fast computational efficiency (speed = 11.920875 Mbit/s). Theoretical analyses and experimental results prove that the proposed scheme has strong resistance to various existing attacks and high level of security.

## 1. Introduction

With the widespread application of a digital image, the security of private image information is of great concern. Various mature block encryption methods are proposed, such as advanced encryption standard (AES) [1,2,3], data encryption standard (DES) [4,5], SM4 [6,7,8] and so on. However, they are not suitable for image encryption due to its redundancy and bi-dimensionality. As a classical permutation method, the Arnold transform [9,10,11,12,13] realizes efficient position swapping, and is widely applied to image encryption field. Hariyanto [9] reveals the effect of Arnold’s iteration number *n* and draws a conclusion that Arnold’s cat map can encrypt the image without reducing the value or information of the digital image. Farwa [10] considers a combination of S-box with a certain number of iterations of the Arnold transform to create confusion and diffusion in the digital image. In the frequency domain, Singh [11] uses a fractional Hartley transform combined with an Arnold transform and singular value decomposition for phase image encryption. However, an obvious weakness is that Arnold transform has periodicity. However, a good encryption algorithm should be not of periodicity. The paper proposes a good method to solve the problem for wide acceptance. First, we calculate the new coordinates for each pixel by the Arnold transform, and then the pixel values are substituted into nonlinear transformation using S-box and linear transformation using chaotic sequences, whose combination is not periodic. Finally, the results are assigned to the new coordinates and get a cipher-image.

Due to the sensitivity of the initial condition, non-periodicity, ergodicity and systematic parameterization, chaotic systems have wide applications in the information security field [14]. The pseudo-random sequence generated by chaotic systems relies heavily on the initial condition. Different initial parameters can yield significantly different pseudo-random sequences. Therefore, it is very difficult to attack a chaotic map by differential attack and statistical analysis. Many interesting image encryption algorithms based on different chaotic maps were proposed in recent years. A piecewise linear chaotic map and a four-dimensional hyper-chaotic map are used to control the permutation and diffusion processes in an algorithm [15]. The algorithm [16] utilizes a Chebyshev map and rotation equation for permutation and substitution operations. The hyper-chaotic Lorenz system in algorithm [17] generates pseudo-random sequences for basic rules of genetic recombination in each phase. As a pseudo-random number generator, quantum chaotic map [18,19,20,21,22] is favored by researchers due to properties of mixing and similar randomness. Liu [21] proposes folding algorithm to XOR each part of the image with quantum chaotic numbers from eight directions and obtains high diffusivity and randomness.

Hash functions [23] are widely utilized in the security layer of every communication protocol and in signature authentication schemes for electronic transactions. The slight alteration of the input parameter can cause massive changes in the output result. In image encryption field, SHA-256 hash of the plain-image is often used to generate secret keys to resist known/chosen-plaintext attack [24,25,26]. A key generator with the plain-image as the input parameter has an obvious weakness: the same security keys are generated when the same image is input. Blockchain technology has become a hot area due to the successful application of Bitcoin [27]. Proofs of work take a block and a string of random numbers as the input parameters of SHA-256 hash function to get a new block. Using random sequences as part of the input parameters can generate different new blocks every time. Inspired by the idea, the plain-image is added into a random sequence for a flexible value and SHA-256 hash computes it to yield security key in the paper. The design not only retains the ability to defend against known/chosen-plaintext attack but also realizes one-time pad [28,29].

The encryption architecture of sole permutation or gray-level encryption is unsafe due to the lack of capacity to resist statistical attack. Generally, the combination of them can obtain better results. Different from common separation architecture, the proposed algorithm carries out permutation and gray-level encryption together. Before the execution of Arnold transform for each pixel, the gray-level encryption is performed by means of S-box substitution [30,31], mutation operation and linear transformation with a quantum chaotic sequence.

The major contributions of the proposed scheme are as follows:

(1) We design an efficient and lossless image encryption scheme based on novel architecture, which combines permutation with scrambling. Before the permutation of the Arnold transform, the gray-level encryption has executed by means of S-box substitution and linear transformation with quantum chaos sequence. The architecture combines permutation and gray-level encryption and eliminates the periodicity brought by the Arnold transform.
(2) Traditional key generator based on SHA-256 hash gets a fixed value if the plain-image has no change. We take a string of random numbers and the plain-image into SHA-256 hash and get a flexible security key due to change in random numbers. This scheme preserves the advantages of SHA-256 hash and provides a flexible security key.
(3) The algebraic transformation Equation (7) is designed to get some extremely sensitive variables to session keys for resistance against key sensitivity attacks. A slight alteration of security key can cause unpredictable changes in the initial parameters and conditions.
(4) Mutation operation based on quantum random selection is presented to modify the value of diffused pixels for high randomness. The results prove that it can decrease the relationship of adjacent pixels in multiple directions.

The rest of this paper is organized as follows. Section 2 introduces the basic theory of the proposed scheme. Section 3 includes encryption and decryption. Section 4 analyzes the level of security. The conclusion is drawn in Section 5.

## 2. Preliminary

### 2.1. General Arnold Transform with Keys

Arnold transform [9,10,11,12,13], also called cat map transform, is utilized as a classical tool to disturb the high correlation among pixels. The definition of general two-dimensional Arnold transform is given as follows.

(1) $$\left[\begin{array}{c} x^{\prime }\\ y^{\prime } \end{array}\right]=A^{n}\left[\begin{array}{c} x\\ y \end{array}\right](\mathrm{mod}N),A=\left[\begin{array}{cc} 1 & a\\ b & ab+1 \end{array}\right]$$

where *n* is iteration times of the matrix *A*. The pixel coordinate of the *N* × *N* plain-image ${\left(x,y\right)}^{T}$ becomes ${\left(x^{\prime },y^{\prime }\right)}^{T}$ of the cipher-image under general Arnold transform. Considering the result that orthogonal transformation is a limited discrete set, we add secret keys ${\left(ku,kv\right)}^{T}$ to get high shuffling and enlarge the key space. The definition of general two-dimensional Arnold transform with keys is given below.

(2) $$\left[\begin{array}{c} x^{\prime }\\ y^{\prime } \end{array}\right]=A^{n}\left[\begin{array}{c} x\\ y \end{array}\right]+\left[\begin{array}{c} ku\\ kv \end{array}\right](\mathrm{mod}N),A=\left[\begin{array}{cc} 1 & a\\ b & ab+1 \end{array}\right].$$

The inverse transformation of Equation (2) is shown as follows.

(3) $$\left[\begin{array}{c} x\\ y \end{array}\right]=A^{-n}\left[\begin{array}{c} x^{\prime }-ku+N\\ y^{\prime }-kv+N \end{array}\right](\mathrm{mod}N),A^{-1}=\left[\begin{array}{cc} ab+1 & -a\\ -b & 1 \end{array}\right].$$

### 2.2. Quantum Chaotic Map

The primary question of quantum chaos is to find the relationship between quantum mechanics and classical chaos. Dissipative quantum systems are coupled to a path of harmonic oscillators to cause quantum logistic map [18,19,20,21,22] with quantum corrections α=<α>+δα, where *δα* shows a quantum fluctuation about <*α*>. Akhshani [22] proves that the very lowest-order quantum can yield a chaotic map as follows.

(4) $$\begin{array}{l} \phi ({x^{\prime }}_{n})=\;r\;({x^{\prime }}_{n}-|{x^{\prime }}_{n}{|}^{2})-r\;{y^{\prime }}_{n}\\ \phi ({y^{\prime }}_{n})=\;-{y^{\prime }}_{n}e^{-2\beta }{+\;e}^{-\beta }r\;[(2-{x^{\prime }}_{n}-{x^{\prime }}_{n}{}^{*}){y^{\prime }}_{n}-{x^{\prime }}_{n}{z^{\prime }}_{n}{}^{*}-{x^{\prime }}_{n}{}^{*}{z^{\prime }}_{n}]\\ \phi ({z^{\prime }}_{n})=\;-{z^{\prime }}_{n}e^{-2\beta }{+\;e}^{-\beta }r\;[2(1-{x^{\prime }}_{n}^{*})\;{z^{\prime }}_{n}-2{x^{\prime }}_{n}{y^{\prime }}_{n}-{x^{\prime }}_{n}] \end{array}$$

where $x^{\prime }=<\alpha >$, $y^{\prime }=$ *<δα* + *δα>*, $z^{\prime }=$ *<δα δα>* and the notation < > represents the expectation value. *β* is the dissipation parameter and *r* is the control parameter. ${x^{\prime }}_{n}^{*}$ is the complex conjugate of ${x^{\prime }}_{n}$ and similarly for ${z^{\prime }}_{n}^{*}$. The range of the parameters as follows: $0\leq {x^{\prime }}_{n}\leq 1$, $0\leq {y^{\prime }}_{n}\leq 0.1$, $0\leq {z^{\prime }}_{n}\leq 0.2$, β∈[6, +∞], r∈[0, 4]. We set r=3.99, β=6 according to Reference [20] and the initial parameters to be real. Iterating Equation (4), the required pseudo-random sequences, based on the quantum chaotic map, are produced.

### 2.3. Mutation Operation

Genetic algorithms are commonly used to generate high-quality solutions to optimization and search problems by relying on bio-inspired operators such as mutation, crossover and selection [32,33]. In biology, mutation is the permanent alteration of the genome’s nucleotide sequence in an organism, virus, or extrachromosomal DNA or other genetic elements. Mutation results in various types of change in sequences. As one of the bio-inspired operators, mutation operation changes two bits of each pixel at a certain rule based on the quantum logistic map. The definition of mutation operation is given below:

(5) gm=bitset(pm,bt,vl)

where *gm* is an offspring value. bitset(pm,bt,vl) is a function to set bit position *bt* in *pm* to *vl*. bt=1,2,…,8 is a pseudo-random number based on the quantum logistic map. If the value of bit position *bt* in *pm* is 1, then sets *vl* = 0, otherwise *vl* = 1. 

## 3. Cryptosystem

**Figure 1:**
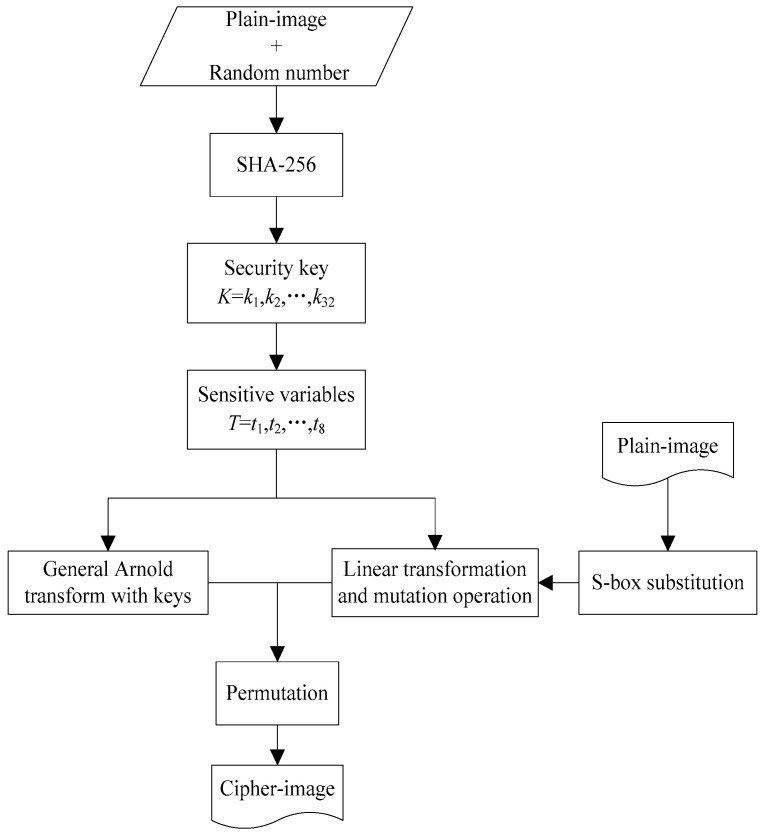
Overall architecture of the proposed cryptosystem.

The proposed cryptosystem includes two stages. In the first stage, keystreams are produced by the key generator based on SHA-256 hash with the input of the plain-image added a random sequence. In the second stage, the permutation and gray-level encryption structure is built by discrete Arnold map combined with S-box substitution, linear transformation and mutation operation. The architecture of the proposed cryptosystem is shown in Figure 1.

### 3.1. Key Generator

Key generator based on SHA-256 hash is a tool to generate security key *K*, which is divided into 8-bit blocks, *k_i_*, referred to as session keys. The cryptosystem converts the plain-image to an array, which is followed by eight random integers ranging from 0 to 255. Inputting the array into SHA-256 hash, the cryptosystem gets a 256-bit binary number as a security key, which is enough large key space to resist any brute-force attack. If one bit of the plain-image toggles, the output result of the SHA-256 hash will have total changes. This approach has a strong ability to defend against known/chosen-plaintext attack. Every execution of key generator can generate different random integers, which causes the different output results of SHA-256 hash and realizes a one-time pad. The 256-bit security key is given as

(6) $$K=\;k_{1},k_{2},\ldots ,k_{32}.$$

Taking key sensitivity into consideration, the paper designs the algebraic transformation to get some extremely sensitive variables to session keys as follows.

(7) $$t_{i}=\left(\left(\frac{k_{i}}{k_{i+8}+1}+\frac{k_{i+8}}{k_{i+16}+1}+\frac{k_{i+16}}{k_{i+24}+1}+\frac{k_{i+24}}{k_{i}+1}\right)\times \sum_{j=1}^{j=32}\frac{k_{j}\times 2^{j-1}}{2^{40}}\right)\mathrm{mod}1$$

where i=1,2,…,8 and $t_{i}\in \left(0,1\right)$. The variables *t_i_* consist of two terms: a variable $\frac{k_{i}}{k_{i+8}+1}+\frac{k_{i+8}}{k_{i+16}+1}+\frac{k_{i+16}}{k_{i+24}+1}+\frac{k_{i+24}}{k_{i}+1}$ for each parameter and a constant $\sum_{j=1}^{j=32}\frac{k_{j}\times 2^{j-1}}{2^{40}}$ for all parameters. When one bit of *K* changes, the values of $\sum_{j=1}^{j=32}\frac{k_{j}\times 2^{j-1}}{2^{40}}$ and also *t_i_* will change. If *k_i_* and *k_i_*_+8_ both alter but the sum does not change, the values of $\frac{k_{i}}{k_{i+8}+1}+\frac{k_{i+8}}{k_{i+16}+1}+\frac{k_{i+16}}{k_{i+24}+1}+\frac{k_{i+24}}{k_{i}+1}$ and $\sum_{j=1}^{j=32}\frac{k_{j}\times 2^{j-1}}{2^{40}}$ change correspondingly. The above statement shows that it is difficult to get the same *t_i_* with different security key *K*. Thus, we conclude that the key generator can resist any key sensitivity attack.

In order to meet the ranges, the initial parameters and conditions of the proposed cryptosystem are derived as follows.

(8) $$\lambda_{i}=\left(\left(\sum_{j=1}^{j=4}(t_{i})-t_{i}\right)\times t_{i}\times 2^{40}\right)\mathrm{mod}256$$

where i=1,2,…,4; initial parameters *a*, *b* and ${\left(ku,kv\right)}^{T}$ of general Arnold transform with keys are set equal to $\lambda_{1}$, $\lambda_{2}$ and ${\left(\lambda_{3},\lambda_{4}\right)}^{T}$. The initial parameters *x*_0_, *y*_0_ and *z*_0_ of the quantum chaotic map are set equal to *t*_5_, $t_{6}\mathrm{mod}0.1$ and $t_{7}\mathrm{mod}0.2$. In order to avoid harmful effects of the quantum chaotic system, the former *m* values are deserted. The initial parameter m∈[500,1500) is defined as follows.

(9) $$m=500+\left(\left(\sum_{i=1}^{i=8}(t_{i})-t_{8}\right)\times t_{8}\times 2^{40}\right)\mathrm{mod}1000.$$

### 3.2. Encryption Approach

The permutation and gray-level encryption structure is built in the encryption stage. First, the new pixel coordinate is calculated by the general Arnold transform with keys. And then the pixel value is modified by S-box substitution combined with linear transform and mutation operation. Finally, the pixel is permuted to the new pixel coordinate. Iterating the process for each pixel, we get the final cipher-image. The encryption algorithm is described as follows.

*Step* 1. Setting L=N×N and substituting the initial parameters *x*_0_, *y*_0_ and *z*_0_ of the quantum chaotic map. Iterating Equation (4) *m* + *L* times and discard the former *m* values to avoid harmful effects. The following equation is applied to get three vectors *Qx*, *Qy* and *Qz* with highly chaotic characteristics.

(10) $$\begin{array}{l} Qx\left(i\right)=\left(x_{i+m}\times N\times N\times 10^{3}\right)\;\mathrm{mod}256\\ Qy\left(i\right)=\left(y_{i+m}\times N\times N\times 10^{3}\right)\;\mathrm{mod}256\\ Qz\left(i\right)=\left(z_{i+m}\times N\times N\times 10^{3}\right)\;\mathrm{mod}256 \end{array}$$

where i=1,2,…,L. Transform vectors *Qx*, *Qy* and *Qz* into three *N* × *N* matrices *Qtx*, *Qty* and *Qtz*. 

Implementing the following XOR operation and get a vector *Qm*.

(11) Qm=Qtx⊕Qty⊕Qtz.

*Step* 2. Substituting the initial parameters *a*, *b* and ${\left(ku,kv\right)}^{T}$ of general Arnold transform with keys. Executing Equation (2), the pixel coordinate ${\left(x,y\right)}^{T}$ of the plain-image becomes ${\left(x^{\prime },y^{\prime }\right)}^{T}$ of the cipher-image.

*Step* 3. The gray-level encryption is executed by S-box substitution and linear transformation as follows.

(12) $$tempe=(S[P(x,y)]⊕Qtx(x^{\prime },y^{\prime })+Qty(x^{\prime },y^{\prime }))⊕Qtz(x^{\prime },y^{\prime })\mathrm{mod}256$$

where x=1,2,…,N, y=1,2,…,N. *P* is the *N* × *N* plain-image. Note that the function S substitutes the value of the current argument according to standard S-box of AES [34]. 

*Step* 4. Two mutation bits of each pixel are pseudo-random based on the quantum matrix *Qm*. The protocol of mutation-selection is given below.

(13) $$\begin{array}{l} mb_{1}=Qm(x,y)\mathrm{mod}8+1\\ mb_{2}=(Qm(x,y)\mathrm{mod}8+4)\mathrm{mod}8+1 \end{array}$$

where *mb*_1_ and *mb*_2_ are mutation bits of the variable *tempe*. Equation (13) is designed for the purpose that two mutation bits are four bits apart. Setting pm=tempe and substituting mutation bits *mb*_1_ and *mb*_2_ into *bt* respectively. Executing Equation (5), the cryptosystem gets an offspring *tempe’*.

*Step* 5. The offspring *tempe’* is assigned to an *N* × *N* matrix $C(x^{\prime },y^{\prime })$.

*Step* 6. Iterating *Steps* 2–5 for each pixel of plain-image, we get the cipher-image *C*. 

### 3.3. Decryption Approach

Decryption approach is easily derived from the encryption routine. First, the inverse transformation of the general Arnold transform with keys is utilized to compute the coordinates. And then the inverse transformation of the gray-level encryption and the same mutation operation are executed to get the pixel value of the plain-image. Finally, the cryptosystem permutes the pixel values to the original coordinate. Iterating the process for each pixel, the cryptosystem realizes the decryption. The decryption algorithm is described as follows.

*Step* 1. The 256-bit security key *K* is substituted into Equations (7)–(9) for the initial parameters and conditions according to Section 3.1.

*Step* 2. By Executing *Step* 1 in Section 3.2, we obtain four *N* × *N* matrices *Qtx*, *Qty*, *Qtz* and *Qm* with highly chaotic characteristics.

*Step* 3. Substituting the initial parameters *a*, *b* and ${\left(ku,kv\right)}^{T}$ to Equation (3) and executing Equation (3), the pixel coordinate ${\left(x^{\prime },y^{\prime }\right)}^{T}$ of the cipher-image *C* becomes ${\left(x,y\right)}^{T}$.

*Step* 4. The same protocol of mutation selection is given in Equation (13). Setting $pm=C(x^{\prime },y^{\prime })$ and substituting mutation bits *mb*_1_, *mb*_2_ into *bt* respectively, the cryptosystem executes Equation (5) for the offspring *tempd*. 

*Step* 5. The decryption method of Equation (14) is shown as follows.

(14) $$tempd^{\prime }=\mathrm{InvS}[((tempd⊕Qtz(x^{\prime },y^{\prime })-Qty(x^{\prime },y^{\prime })+256)\mathrm{mod}256)⊕Qtx(x^{\prime },y^{\prime })]$$

where x=1,2,…,N, y=1,2,…,N. Note that the function InvS substitutes the value of the current argument according to inverse S-box of AES [34].

*Step* 6. The result *tempe’* of Equation (14) is assigned to an *N* × *N* matrix I(x,y).

*Step* 7. Iterating *Steps* 3–6 for each pixel of the cipher-image, we get decrypted matrix *I*, which is the same as the plain-image *P*.

## 4. Security Analysis

In this section, several experiments are conducted to check the security of the proposed cryptosystem, including histogram analysis, correlation coefficients, information entropy, differential analysis, key sensitivity analysis and key space analysis. The paper selects four standard gray-level images (256 × 256 Lena, 256 × 256 Cameraman, 512 × 512 Baboon and 512 × 512 Boats) from CVG-UGR image database for tests.

### 4.1. Histogram Analysis

**Figure 2:**
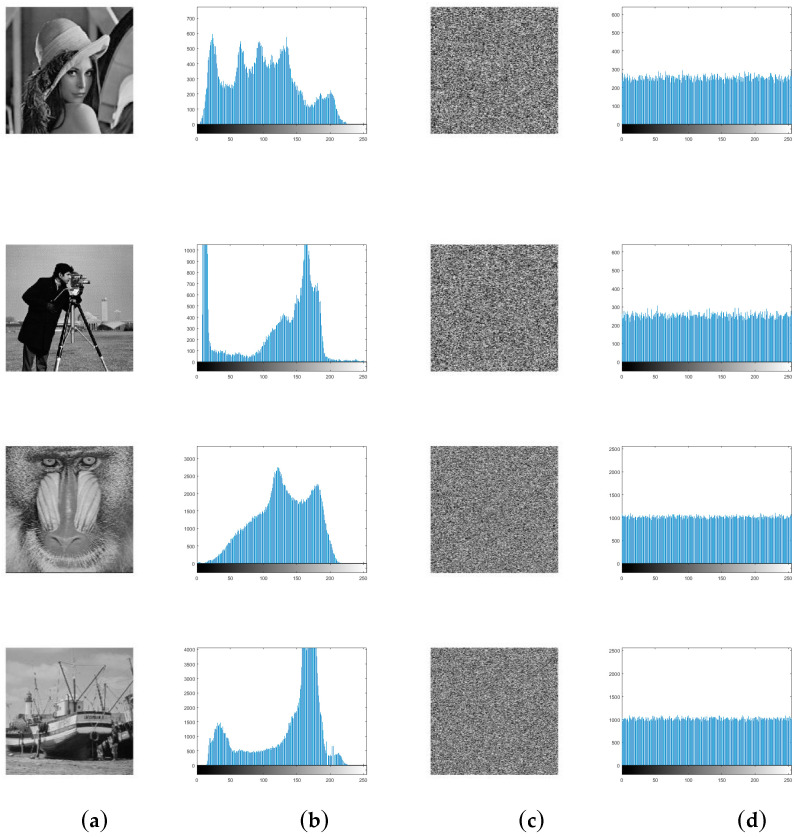
(**a**) plain-images; (**b**) histograms of the plain-images; (**c**) cipher-images; (**d**) histograms of the cipher-images.

Histogram reveals the distribution of pixel intensity values in an image. An ideal cipher-image is of a uniform frequency distribution that does not provide attack opponents with any useful statistical information. It is obvious that the distributions of plain-images are concentrated on some values while those of cipher-images are fairly uniform in Figure 2.

The variance of the histogram is an important parameter to measure the uniformity of the distribution of pixel values. The lower variance means the higher uniformity of histogram. The variance [35] of the histogram is defined as follows:

(15) $$\mathrm{var}(Z)=\frac{1}{256^{2}}\sum_{i=1}^{256}\sum_{j=1}^{256}\frac{1}{2}{\left(z_{i}-z_{j}\right)}^{2}$$

where *Z* is the vector of the histogram values and *Z* = {*z*_1_, *z*_2_, …, *z*_256_}. *z_i_* and *z_j_* are the numbers of pixels which values are equal to *i* and *j* respectively. Table 1 shows the variances of plain-images and cipher-images. By comparing results in Table 1, we can see that variances of cipher-images are much smaller than those of plain-images. Thus, we can conclude that the proposed algorithm has the ability to defend the histogram analysis.

### 4.2. Correlation Coefficients

**Figure 3:**
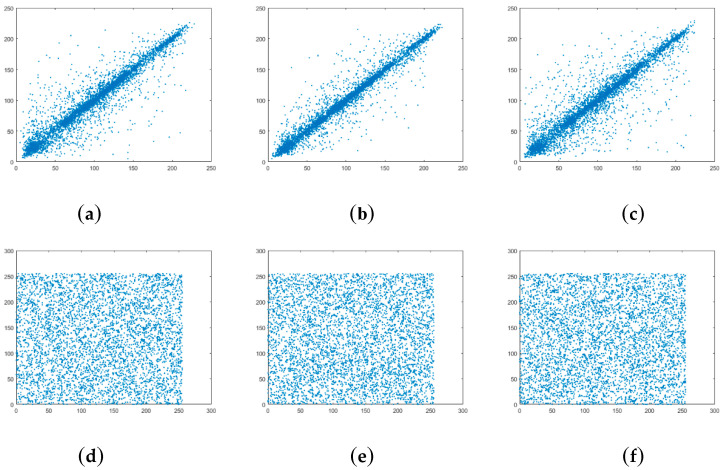
Correlation of two adjacent pixels in the plain-image of 256 × 256 Lena: (**a**) horizontal direction; (**b**) vertical direction; (**c**) diagonal direction. Correlation of two adjacent pixels in the cipher-image of 256 × 256 Lena: (**d**) horizontal direction; (**e**) vertical direction; (**f**) diagonal direction.

One of the most important differences between the image and the text is the correlation of two adjacent digital information. Considering the two-dimensional nature of the image, the correlation comes from horizontal, vertical and diagonal directions. The normal image is of high correlation and the cipher-image encrypted by good encryption schemes should have low correlation. The paper randomly selects 5000 pairs of two adjacent pixels from the plain-image 256 × 256 Lena and its cipher-image for simulation tests and calculates the correlation coefficients as follows.

(16) $$\begin{array}{l} E=\frac{1}{N}\sum_{i=1}^{N}w_{i}\\ D(w)=\frac{1}{N}\sum_{i=1}^{N}{\left(w_{i}-E(w)\right)}^{2}\\ Cov(w,z)=\frac{1}{N}\sum_{i=1}^{N}\left(w_{i}-E(w))(z_{i}-E(z)\right)\\ r_{wz}=\frac{Cov(w,z)}{\sqrt{D(w)}\sqrt{D(z)}} \end{array}$$

where *w*, *z* are gray values of adjacent pixels. *r_wz_* is correlation coefficients of the image. Figure 3 shows distributions of correlation along horizontal, vertical and diagonal directions for 256 × 256 Lena. Correlation coefficients along horizontal, vertical and diagonal direction are reported in Table 2.

### 4.3. Information Entropy

Information entropy describes the average rate at which information is produced by a stochastic source of data. It is applied to evaluate the uncertainty and degree of ambiguity in the image. The mathematical definition of entropy is given below.

(17) $$H(s)=-\sum_{i=0}^{2^{n}-1}p(s_{i})\log_{2}p(s_{i})$$

where *s* is the amount of gray level in the image and *p*(*s_i_*) is the probability of the symbol *s_i_*. The ideal value of information entropy is very close to 8 for the cipher-image with 2^8^ gray-levels. Table 3 reports entropy values of four standard images and their cipher-images using the proposed scheme. Table 4 shows the comparison of information entropy for the proposed scheme and different schemes in the 256 × 256 image Lena. The results of information entropy are very close to the ideal value 8 and manifest better randomness and uncertainty than other algorithms.

### 4.4. Sensitivity Analysis

#### 4.4.1. Differential Analysis

Differential analysis is a method to assess sensitivity to the plain-image. An ability to resist differential attack means that a slight change in the plain-image causes significant changes in the cipher-image. There are two scales for measuring the differences between two cipher-images: number of pixels change rate (*NPCR*) using Equation (19) and unified average changing intensity (*UACI*) using Equation (20).

(18) $$D(i,j)=\{\begin{array}{ll} D(i,j)=0, & C(i,j)=C(i,j{)}^{\prime }\\ D(i,j)=1, & C(i,j)\neq C(i,j{)}^{\prime } \end{array}$$

(19) $$NPCR=\frac{\sum {}_{i,j}D(i,j)}{N\times N}\times 100\%$$

(20) $$UACI=\frac{1}{N\times N}\left[\sum_{i,j}\frac{\left|C(i,j)-C(i,j{)}^{\prime }\right|}{255}\right]\times 100\%$$

where *N* × *N* cipher-images *C* and *C’* are results of the proposed scheme with different plain-images in only one pixel. Under ideal conditions, NPCR=99.609375% and UACI=33.463542%. One bit is added to a random pixel value of the plain-image. Results of *NPCR* and *UACI* are calculated for four standard images and reported in Table 5. Table 6 reports *NPCR* and *UACI* for 1-bit change of the plain-image Lena in different pixels and Table 7 compares the proposed scheme with other schemes for the plain-image sensitivity test. The results show that the proposed scheme has a strong ability to resist known/chosen-plaintext attack.

#### 4.4.2. Security Key Analysis

**Figure 4:**
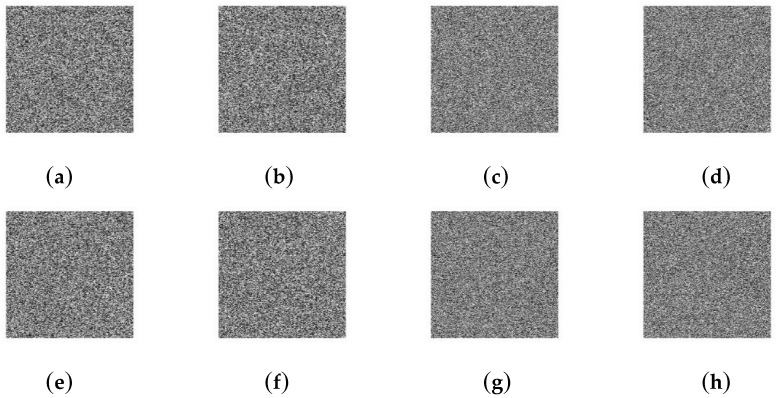
Encrypt with *K*: (**a**) Lena; (**b**) Cameraman; (**c**) Baboon; (**d**) Boats. Decrypt with *K’*: (**e**) Lena; (**f**) Cameraman; (**g**) Baboon; (**h**) Boats.

Security key analysis reveals the sensitivity to the security key. Usually, we consider key sensitivity in two aspects. One is that a minor change in security key yields significant differences in cipher-images; the other is that the attacker cannot decrypt the cipher-image with other keys. Even if these keys are only slightly different from the security key. The paper encrypts four standard images with different keys and measures the differences in terms of *NPCR* and *UACI*. Table 8 reports the significant differences between two cipher-images when security key changes from *K* = 227, 48, 167, 206, 231, 63, 103, 182, 95, 180, 90, 116, 145, 164, 5, 178, 55, 207, 152, 140, 143, 215, 109, 203, 139, 204, 70, 23, 165, 223, 138, 144 to *K’* = 226, 48, 167, 206, 231, 63, 103, 182, 95, 180, 90, 116, 145, 164, 5, 178, 55, 207, 152, 140, 143, 215, 109, 203, 139, 204, 70, 23, 165, 223, 138, 144. We encrypt four standard images with *K* and then decrypt them with *K’*. Figure 4 shows that a slight change of security key cannot decrypt the cipher-image.

### 4.5. Mean Squared Error

Mean squared error (*MSE*) reflects the degree of difference between the two variables. The paper uses *MSE* to measure the differences between the *N* × *N* plain-image *P*(*i*, *j*) and its cipher-image *C*(*i*, *j*). *MSE* is calculated using the following equation [37].

(21) $$MSE=\frac{1}{N\times N}\sum_{j=1}^{N}\sum_{i=1}^{N}{\left|C(i,j)-P(i,j)\right|}^{2}.$$

### 4.6. Root Mean Squared Error

In order to discover the precision of the encryption scheme, root mean squared error (*RMSE*) is applied to compute the error between the plain-image and its cipher-image [38].

(22) $$RMSE=\sqrt{\frac{1}{N\times N}\sum_{j=1}^{N}\sum_{i=1}^{N}{\left|C(i,j)-P(i,j)\right|}^{2}}$$

where *P*(*i*, *j*) is the pixel intensity of the *N* × *N* plain-image and the *C*(*i*, *j*) is the pixel intensity of the *N* × *N* cipher-image. 

### 4.7. Mean Absolute Error

Mean absolute error (*MAE*) is a parameter to measure the differences between two continuous variables in statistics. *MAE* is figured to evaluate how the cipher-image *C*(*i*, *j*) is not the same as the plain-image *P*(*i*, *j*). *MAE* is defined as [36,37].

(23) $$MAE=\frac{1}{N\times N}\sum_{j=1}^{N}\sum_{i=1}^{N}\left|C(i,j)-P(i,j)\right|.$$

The larger *MAE* indicates the higher security level. The paper calculates *MSE*, *RMSE* and *MAE* values for four standard images and shows in Table 9.

### 4.8. Key Space Analysis

Brute-force attack consists of an attacker trying all possible security keys by an exhaustive key search until the correct one is found. Therefore, small key space cannot resist brute-force attack. For achieving high resistance against a brute-force attack, the size of the key space should be larger than 2^128^ [39,40]. Security key of the proposed algorithm is generated by SHA-256 hash, whose output result is 256-bit long. The size of the key space is 2^256^ > 2^128^. So we claim that key space is enough large to resist all kinds of brute-force.

### 4.9. Running Performance

Apart from security considerations, efficiency is also an important parameter for a cryptosystem. The actual execution time depends on many factors including CPU performance, memory size, programming skill. The simulation is implemented on MATLAB R2015b in a computer of an Intel Core I3 CPU 2.30 GHz and 4 GB of RAM. We execute the encryption process for 512 × 512 images 500 times and calculate the total time 83.886457 s. Thus, the real encryption efficiency is $efficiency=\frac{512\times 512\times 8\times 500}{2^{20}\times 83.886457}\mathrm{Mbit}/s=11.920875\mathrm{Mbit}/s$. The result shows the proposed cryptosystem has fast running performance.

## 5. Conclusions

The paper designs a novel image encryption scheme based on general Arnold transform with keys combined with S-box and mutation operation. Key generator using SHA-256 hash aims to generate security key and the initial parameters of Arnold map and quantum chaotic map. SHA-256 hash with the plain-image and random numbers can yield a 256-bit output as a security key. If the plain-image is altered, security key changes corresponding. So the proposed scheme can resist differential attack and known/chosen-plaintext attack. When we encrypt the same plain-image more than once, the change of random numbers will affect the output result of SHA-256 hash. The classical Arnold map is a practical tool to permute the pixel. The paper applies general Arnold transform with keys for the higher scrambling. In order to get high complexity and randomness, the paper proposes a more complicated architecture, which combines permutation with gray-level encryption. S-box is introduced into the encryption scheme as a nonlinear transformation and integrated with linear transformation and mutation operation to decrease the correlation of two adjacent pixels. The cipher-image from the proposed cryptosystem has fairly uniform histogram, low correlation coefficients closed to 0, high information entropy closed to 8. The proposed cryptosystem provides 2^256^ key space and performs fast computational speed (*efficiency* = 11.920875 Mbit/s). The experimental results demonstrate that the proposed scheme achieves high sensitivity to security key and the plain-image, low correlation coefficients, ideal information entropy, large key space and strong resistance against all kinds of attacks.

## Figures and Tables

**Table 1:** Variances of histogram between plain-image and cipher-image.

Images	Lena	Cameraman	Baboon	Boats
Plain-image	30665.70	110973.30	750395.63	1583351.60
Cipher-image	256.54	251.85	975.09	931.88

**Table 2:** Correlation coefficients of pair adjacent pixels in plain and cipher images.

Scan Direction	Lena		Cameraman		Baboon		Boats	
	Plain	Cipher	Plain	Cipher	Plain	Cipher	Plain	Cipher
Horizontal	0.938596	0.000455	0.926835	0.000282	0.839511	0.001276	0.808009	0.000560
Vertical	0.966742	0.002175	0.958801	0.016975	0.724123	0.021980	0.961392	0.002198
Diagonal	0.916518	0.003801	0.912749	0.011328	0.684117	0.010744	0.833386	0.010743

**Table 3:** Information entropy.

Images	Lena	Cameraman	Baboon	Boats
Plain-image	7.568285	7.009716	7.357949	7.123758
Cipher-image	7.997860	7.997538	7.999354	7.999332

**Table 4:** Comparison of information entropy for the proposed scheme and different schemes.

Algorithm	Information Entropy
Proposed	7.9979
[21]	7.9973
[24]	7.9973
[36]	7.9972

**Table 5:** Average *NPCR* and *UACI* for the plain-image sensitivity test.

Images	*NPCR*	*UACI*
Lena	0.996119	0.334033
Cameraman	0.996017	0.334313
Baboon	0.996094	0.334526
Boats	0.996017	0.334361

**Table 6:** The image Lena for the plain-image sensitivity test in different pixels.

Pixels	(1, 1)	(27, 103)	(144, 178)	(201, 224)	(217,105)	(255,255)
*NPCR*	0.996383	0.996199	0.996262	0.996093	0.996338	0.995834
*UACI*	0.334751	0.333843	0.334560	0.333072	0.334543	0.333060

**Table 7:** Comparison of the plain-image sensitivity test in the image Lena.

Algorithm	*NPCR*	*UACI*
Proposed	0.996119	0.334033
[20]	0.996012	0.335376
[24]	0.996074	0.309976
[36]	0.996124	0.334591

**Table 8:** Average *NPCR* and *UACI* for the key sensitivity test.

Images	*NPCR*	*UACI*
Lena	0.996094	0.334603
Cameraman	0.995804	0.336895
Baboon	0.996036	0.334515
Boats	0.996017	0.334361

**Table 9:** *MSE*, *RMSE* and *MAE* between the plain-image and its cipher-image.

Images	Lena	Cameraman	Baboon	Boats
*MSE*	9069.366196	9486.689178	7247.521557	8287.326183
*RMSE*	95.233220	97.399636	85.132377	91.034753
*MAE*	78.201950	79.741455	70.969791	75.038555
